# Impact of gastrointestinal parasitic nematodes of sheep, and the role of advanced molecular tools for exploring epidemiology and drug resistance - an Australian perspective

**DOI:** 10.1186/1756-3305-6-153

**Published:** 2013-05-27

**Authors:** Florian Roeber, Aaron R Jex, Robin B Gasser

**Affiliations:** 1 The University of Melbourne, Victoria 3010, Australia

**Keywords:** Australia, Gastrointestinal nematodes, Strongylida, Small ruminants (including sheep and goats), Molecular methods, Epidemiology, Drug resistance

## Abstract

Parasitic nematodes (roundworms) of small ruminants and other livestock have major economic impacts worldwide. Despite the impact of the diseases caused by these nematodes and the discovery of new therapeutic agents (anthelmintics), there has been relatively limited progress in the development of practical molecular tools to study the epidemiology of these nematodes. Specific diagnosis underpins parasite control, and the detection and monitoring of anthelmintic resistance in livestock parasites, presently a major concern around the world. The purpose of the present article is to provide a concise account of the biology and knowledge of the epidemiology of the gastrointestinal nematodes (order Strongylida), from an Australian perspective, and to emphasize the importance of utilizing advanced molecular tools for the specific diagnosis of nematode infections for refined investigations of parasite epidemiology and drug resistance detection in combination with conventional methods. It also gives a perspective on the possibility of harnessing genetic, genomic and bioinformatic technologies to better understand parasites and control parasitic diseases.

## Review

### Introduction

Parasites of livestock cause diseases of major socio-economic importance worldwide. The current financial and agriculture losses caused by parasites have a substantial impact on farm profitability. For example, the annual cost associated with parasitic diseases in sheep and cattle in Australia has been estimated at 1 billion dollars [1,2] and are proposed to be tens of billions of dollars worldwide, according to the sales of anti-parasitic compounds by pharmaceutical companies, excluding production losses. Thus, there are major economic gains to be made in agriculture by enhancing the control of key parasitic diseases.

Parasitic nematodes of livestock are controlled mainly through anthelmintic treatment. Even with optimally timed, strategic treatments, this type of control is expensive and, in most cases, only partially effective. In addition, the excessive and frequent use of anthelmintics has resulted in substantial and widespread problems with anthelminthic resistance in nematode populations [3-5]. Therefore, there is an obvious need for and significant global interest in the development of improved means of controlling parasitic nematodes. Given the significant problems with drug resistance, there is an urgent need for an increased focus on understanding the epidemiology of parasites, in order to work toward better strategic and integrated approaches of parasite control. The purpose of the present article is to provide a concise account of the biology and current knowledge of the epidemiology of gastrointestinal nematodes in small ruminants in Australia, and to emphasize the importance of the application of advanced molecular tools for the diagnosis of infections for refined investigations into the epidemiology of parasites and drug resistance.

### Key parasitic nematodes of small ruminants

**Figure 1:**
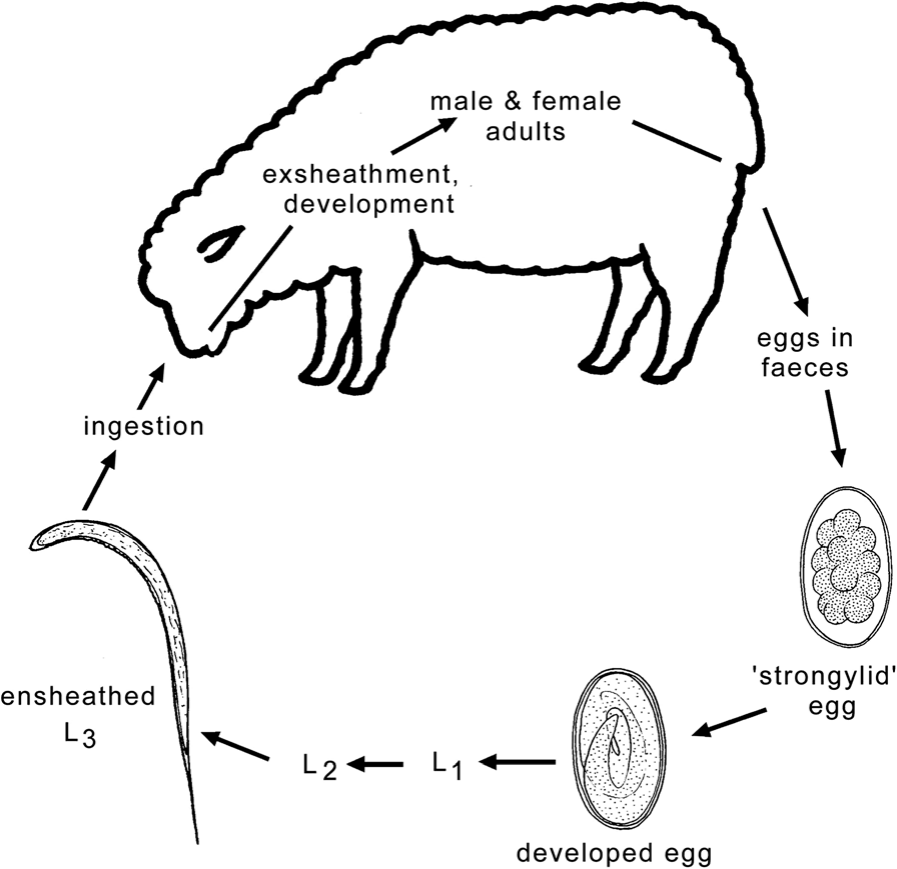
**Life cycle representing gastrointestinal nematodes (order Strongylida) of small ruminants; adapted from **[103]**. **First-, second- and third-stage larvae (L1, L2 and L3, respectively) are free-living in the environment. The fourth larval (L4) and adult stages (dioecious) are parasitic in the gastrointestinal tract of the host. Disease is caused by the L4 and/or adult stages and depends on factors including: species of nematode infecting the host; intensity of the infection; species, age and immunological/health status of the host; host response against the parasite; environment and management aspects [11,15].

The principal gastrointestinal nematodes infecting and affecting small ruminants (sheep and goats) in Australia are *H. contortus*, *Te. circumcincta* and *Trichostrongylus* spp. (Table 1). They belong to the order Strongylida [6]. The life cycles of these nematodes follow a similar pattern, with some exceptions (e.g., *Nematodirus* spp., for which larval development occurs within the egg) (see Figure 1) [7]. Sexually dimorphic adults are present in the digestive tract, where fertilized females produce large numbers of eggs that are passed in the faeces. Strongylid eggs (70–150 μm) usually hatch within 1–2 days. After hatching, larvae feed on bacteria and undergo two moults to then develop to ensheathed third-stage larvae (L3s) in the environment (i.e., faeces or soil). The sheath (which represents the cuticular layer shed in the transition from the L2 to L3 stage) protects the L3 stage from environmental conditions but prevents it from feeding. Infection of the host occurs by ingestion of L3s. During its passage through the stomach, the L3 stage loses its protective sheath and has a histotropic phase (tissue phase), depending on species, prior to its transition to the L4 and pre-adult stages [7]. Under unfavourable conditions (usually at the end of the grazing season), the larvae undergo a period of arrested development, called hypobiosis (typical for species of *Haemonchus* and *Teladorsagia*). Hypobiotic larvae then resume their activity and development in the following spring in the case of *Haemonchus* or autumn in the case of *Teladorsagia*. This may be in synchrony with the beginning of the lambing season, which manifests itself in a peri-parturient increase in faecal egg counts (FECs) in ewes [8]. The peri-parturient decrease of immunity increases the survival and egg production of existing parasites, increases susceptibility to further infections, and contributes to the contamination of pasture with L3s when young, susceptible animals begin grazing [9].

**Table 1:** **Morphological characteristics, pre-patent periods and locations in the host of key genera and species of gastrointestinal nematodes infecting sheep in Australia**[6,7,15,92]

**Family**	**Species**	**Morphometrics/morphology**		**Prepatent period**	**Location in the host**
		**Length (mm)**	**Features**	**(days)**	
Trichostrongylidae	*Haemonchus contortus*	♂ 10-20	Red pseudocoelomic fluid and white coiled ovaries giving the appearance of a barber's pole.	18-21	Abomasum
		♀ 18-30			
			Presence of vulvar flap depends on strain.		
	*Teladorsagia circumcincta*	♂ 7-8	Small head and buccal cavity.	15-21	Abomasum
		♀ 10-12	In females, a vulvar flap can be present.		
	*Trichostrongylus axei*	♂ 2-6	Dissimilar spicules of unequal length.	15-23	Abomasum
		♀ 3-8			or stomach
	*T. colubriformis*	♂ 4-8	Equal length spicules with triangular tip.	15-23	Anterior small intestine
		♀ 5-9			
	*T. vitrinus*	♂ 4-7	Equal length spicules with sharp tips.	15-23	Anterior small intestine
		♀ 5-8			
	*T. rugatus*	♂ 4-7	Dissimilar spicules with foot-like appearance.	15-23	Small intestine
		♀ 6-7			
	*Cooperia curticei*	♂ 4-5	Transverse striation of cuticle in all species.	14-15	Small intestine
		♀ 5-6	Watch-spring-like body posture and presence		
			a small cephalic vesicle are characteristic.		
Molineidae	*Nematodirus spathiger*	♂ 10-19	Small but distinct cephalic vesicle.	18	Small intestine
		♀ 15-29	Very long spicules ending in a		
			spoon-shaped terminal piece.		
	*N. filicollis*	♂ 10-15	Small but distinct cephalic vesicle.	18	Small intestine
		♀ 15-20	Long and slender spicules with a		
			narrow, lanceolate membrane.		
Ancylostomatidae	*Bunostomum trigonocephalum*	♂ 12-17	Anterior end is bend dorsally,	40-70	Small intestine
		♀ 19-26	Buccal capsule with two cutting plates.		
Chabertiidae	*Oesophagostomum columbianum*	♂ 12-16	Have two leaf crowns and a shallow	40-45	Large Intestine
		♀ 14-18	buccal capsule. Position of cervical papillae		
			used for species differentiation.		
	*O. venulosum*	♂ 11-16	Cervical papillae are situated	40-45	Large intestine
		♀ 13-24	posterior to the oesophagus.		
	*Chabertia ovina*	♂ 13-14	Mouth is directed antero-ventrally.	42-50	Large intestine
		♀ 17-20	Buccal capsule is subglobular		
			without teeth.		

Almost all sheep are infected with one or more of these nematodes, but the intensity of infection and clinical signs associated with disease can vary considerably [10]. The severity of disease is mainly influenced by factors such as the parasite species present, the number of worms present in the gastrointestinal tract, the general health and immunological state of the host, and environmental factors, such as climate and pasture type, stress, stocking rate, management and/or diet [11]. Usually, three groups of animals are prone to heavy worm burdens; (i) young, non-immune animals, (ii) adult, immuno-compromised animals, and (iii) those exposed to a high infection pressure from the L3-contaminated environment [12]. Nematode populations in sheep are generally over-dispersed, with only few sheep carrying heavy worm burdens, whilst the majority of sheep harbour low numbers of worms [13].

#### Haemonchus contortus

*Haemonchus contortus* is one of the most fecund strongylid nematodes; individual females are capable of producing thousands of eggs per day, which can lead to rapid larval pasture contamination and associated outbreaks of haemonchosis [7]. In sheep, the pre-patent period of *Haemonchus* is 18–21 days; adult worms are short-lived, surviving in their hosts for only a few months. The main pathogenic effects are caused by the L4s and adults, which both feed on blood, causing severe anaemia which usually becomes apparent after two weeks of infection [14]. Acute disease is usually dependent on the intensity of infection, and is associated with signs of haemorrhagic anaemia, dark-coloured faeces, oedema, weakness, reduced production of wool and muscle mass, or sometimes sudden death. In cases of chronic disease, decreased food intake, weight loss and anaemia are most commonly observed [11,15]. Unlike many other gastrointestinal parasites, *H. contortus* is not a primary cause of diarrhoea, and its effects on a flock are often not readily detected by routine observation [12].

#### Teladorsagia circumcincta

Females of this species are less fecund than *H. contortus*, with an average egg production of 100–200 eggs per female per day [16]. *Teladorsagia* does not feed on blood, and the main pathogenic effects are caused by its larval stages. Larval development takes place in the gastric glands, leading to nodule formation in the abomasal mucosa and extensive damage to parietal cells, in turn causing a decrease in hydrochloric acid production [17,18]. Subsequently, the increase in abomasal pH causes a failure of pepsinogen to convert to the active form of pepsin, which results in elevated plasma pepsinogen levels and reduced protein digestion. The severity of the infection depends on concurrent infections, nutritional state of the host and also its ability to develop an immunogenic response [19]. Commonly, moderate or subclinical infections occur, causing diarrhoea, poor weight gain, weight loss and reduced wool production [12].

#### *Trichostrongylus* species

Species of this genus represent an important group of parasites of grazing small ruminants. These parasites occur in the small intestine and mainly exert their pathogenic effects in lambs and weaners, but have also been reported to cause significant depression of wool growth in older sheep [10]. In Australia, the three most common species are *T. colubriformis*, *T. vitrinus*, and *T. rugatus*[20]. The main pathogenic effects are caused by the exsheathed L3s of *T. vitrinus*, which burrow between the enterocytes of intestinal villi and lead to the formation of intra-epithelial tunnels [21]. Young nematodes develop in these tunnels 10–12 days following infection. The migration of young adult worms is associated with extensive damage to the duodenal mucosa and with signs of generalised enteritis, including haemorrhage, oedema and plasma protein loss into the intestinal lumen, and subsequent hypoalbuminaemia and hypoproteinaemia [22,23]. Infections with *Trichostrongylus* are often difficult to distinguish from malnutrition in the case of low-intensity infections [15] but, if worms are present in high numbers, cause protracted watery diarrhoea, which stains the fleece of the hindquarters (black scours) [7]. *Trichostrongylus axei*, which inhabits the abomasum, is less common and occurs usually in smaller numbers [10].

### Other species

*Cooperia curticei*, *N. spathiger*, *N. fillicollis*, *N. abnormalis* and *O. venulosum* are common parasites of the small and/or large intestine, whilst *C. ovina* and the hookworm, *B. trigonocephalum*, are less common [12]. Individually, these species have relatively low pathogenicity, but may contribute to parasitic gastroenteritis in grazing small ruminants. *Nematodirus battus* is of particular pathogenic significance in some temperate areas, such as the British Isles, where the mass-hatching of infective L3s occurs during spring, causing disease of young lambs [24]. This species of *Nematodirus* has not been reported for Australia.

### Aspects of the epidemiology of gastrointestinal nematodes of sheep

**Figure 2:**
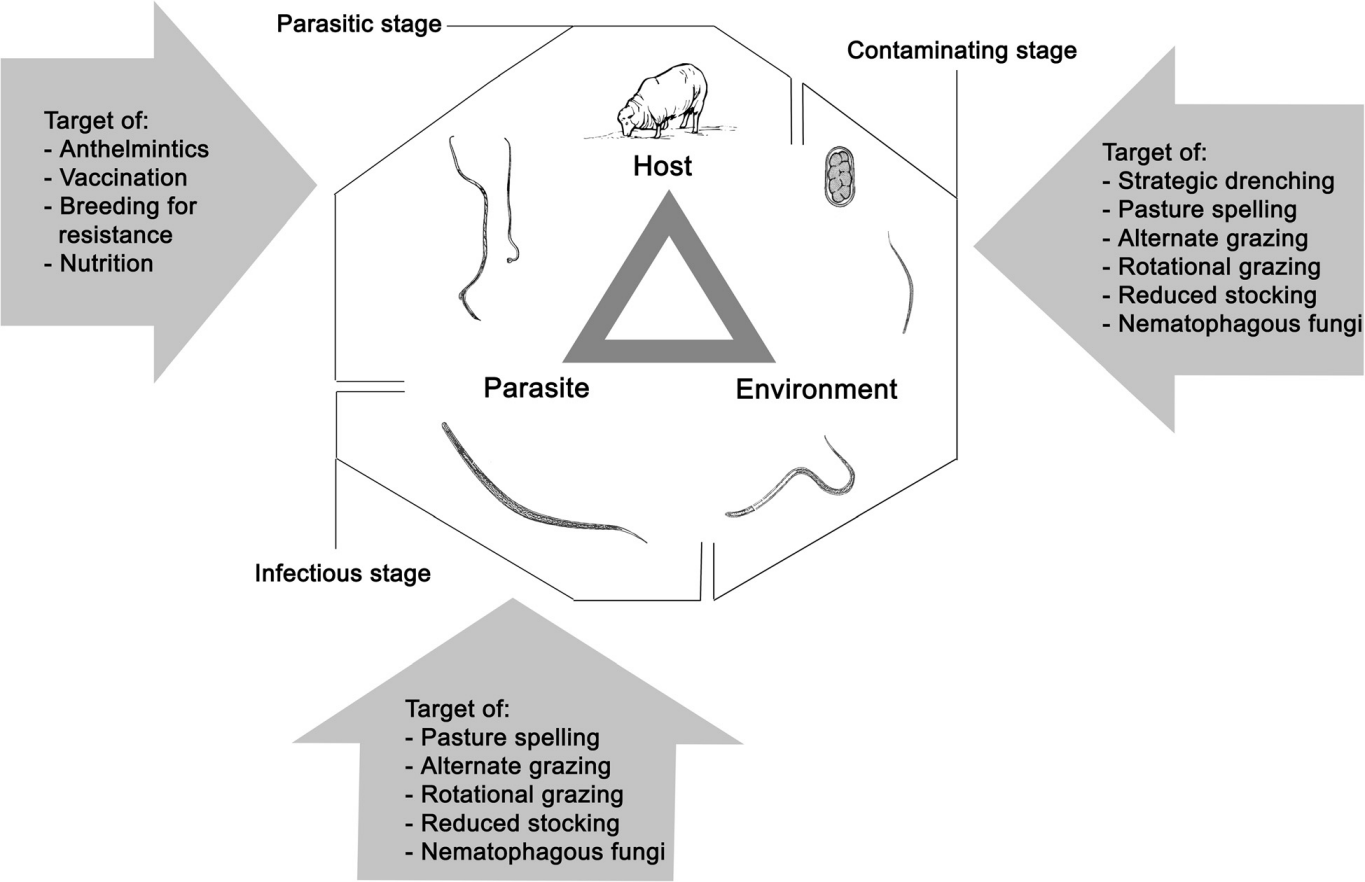
**Relationship among host, parasites and environment, and factors that effect parasite control **[7,][10,][28]**.**

Gastrointestinal nematodes of sheep are transmitted horizontally and directly. The relationship of parasite with host and environment is displayed in Figure 2[7]. Many factors linked to this relationship determine the type and severity of infection. Host-related factors are age, immunity, sex, species and genetic resistance; parasite-related factors include life history, duration of the histotropic phase, survival of larvae in the environment and their location in the host; environmental factors include climate, weather, season, type of vegetation and microclimate. The interactions between host and parasite mainly determine the potential for disease to occur and the pattern/course of infection, whereas the interaction between host-environment and parasite-environment influence disease transmission [7]. Regional differences in climate have major effects on the epidemiology of nematode infections and their geographic distribution [16,20,25]. Because species distributions and the seasonal availability of different parasites are largely determined by their ecological needs (e.g., for the successful development of their free-living stages), environmental conditions, particularly temperature and relative humidity, are of major importance [20,26] (Table 2). Thus, climate impacts directly on the distribution of parasites. However, there are other exogenous factors, such as anthelmintic treatment regimens or host movement, which can influence the distribution and prevalence of these parasitic nematodes in a particular geographic region and environment [27,28]. In spite of many insights into the epidemiology and ecology of parasitic nematodes, there are still significant gaps in our knowledge in these areas for individual species of parasites, mainly due to a lack of accurate diagnostic tools in the past.

**Table 2:** **Main features of major trichostrongylid nematodes of sheep and environmental influences on survival; adapted from**[26]

**Nematode species**	**Life cycle stage**			
	**Unembryonated egg**	**Embryonated egg**	**Pre-infective larvae**	**Infective larvae**
*H. contortus*	High susceptibility to cold and desiccation. High mortality at < 10°C.	Susceptible to cold and desiccation. Low hatching in the abscence of moisture and/or at < 10°C.	High susceptibility to cold and desiccation.	Optimum survival under warm and moist conditions. Poor survival in dry climates (warm or cool) and sub-freezing winter.
*T. colubriformis*	Intermediate susceptibility to cold and desiccation. High mortality at < 5°C.	Intermediate susceptibility to cold. Low susceptibility to desiccation.	Susceptible to cold and desiccation. High mortality at < 5°C.	Optimum survival under warm or cool moist conditions. Poor survival over sub-freezing winters.
*Te. circumcincta*	Low susceptibility to cold. Intermediate susceptibility to desiccation. High egg viability at 0-10°C.	Low susceptibility to cold and desiccation. Hatching at < 5°C.	Intermediate susceptibility to cold. Susceptible to desiccation.	Optimum survival under cool moist conditions and sub-freezing winters. Poor survival under warm, dry conditions.

### Geographical distribution of gastrointestinal nematodes in sheep according to climate zone – the Australian context

**Figure 3:**
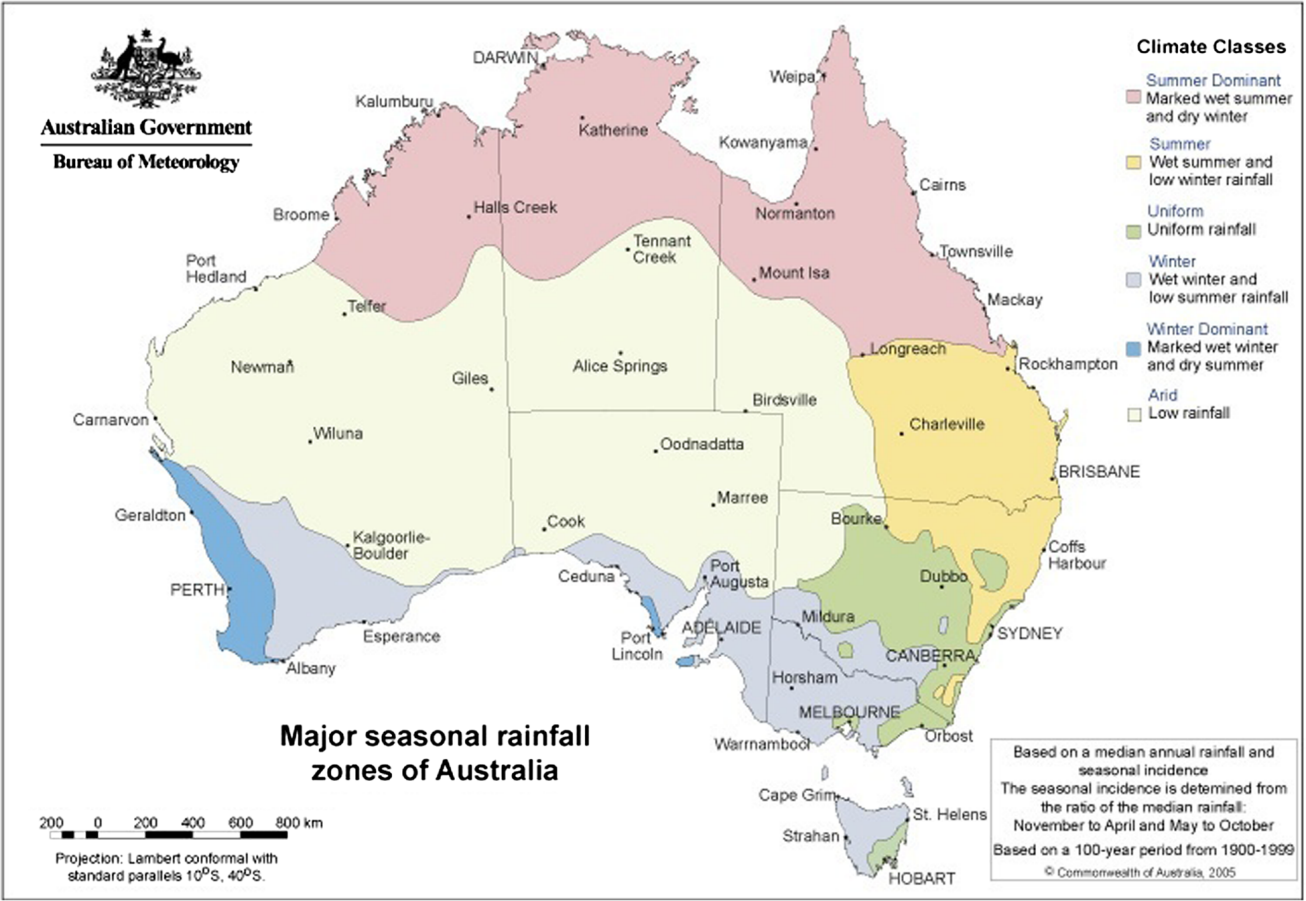
**Map showing the major seasonal rainfall zones of the Australian continent.** Taken from: http://www.bom.gov.au; product code: IDCJCM0000; accession date: 27.10.2011.

The distributions of gastrointestinal nematodes of sheep follow the prevailing seasonal rainfall patterns in areas where sheep are kept [16]. In Australia, three main rainfall zones are recognised: summer, winter and a non-seasonal (uniform) rainfall zone (Figure 3). Most nematodes studied to date occur in all rainfall zones, with the exception of *O. columbianum*, which is absent from winter rainfall zones [10,16]. However, their incidence in different climatic zones varies, and differences can be observed particularly between districts within a zone, being most common in districts with an average annual rainfall of 380 mm or more [16].

For most species of gastrointestinal nematodes, climatic conditions in the winter and uniform rainfall zones during the Australian autumn (April to May) are most suitable, when temperatures and rate of evaporation are moderate to low [10,16]. During spring, rising temperatures create favourable conditions for the development of free-living larvae and enhance their migratory capacity on vegetation. During hot and dry summer conditions, the degree of larval pasture contamination decreases, and larval survival depends largely on rainfall [10]. Falling temperatures during winter slow the development and migratory capacity of nematode larvae but can increase their longevity, so that surviving larvae can be a source of infection in the following spring [10,29]. In summer rainfall zones, development and translation of larvae occur during the wet summer months, while the winter months are either too cold or too dry to permit larval development and translation.

### Summer rainfall zones

**Figure 4:**
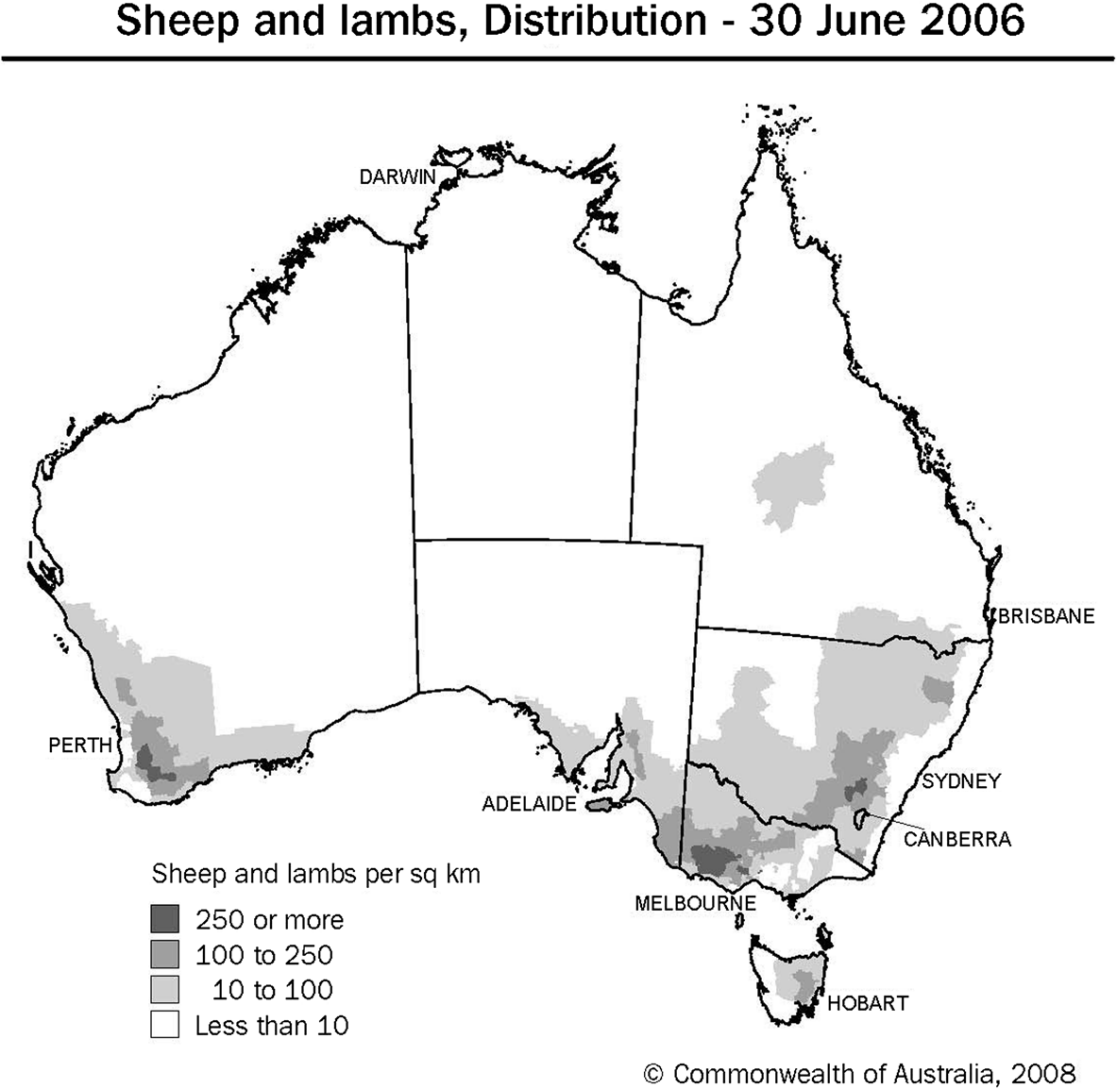
**Distribution of adult sheep and lambs within Australia.** Reference: http://www.abs.gov.au; 7101.0-AgMag-The Agriculture Newsletter.

In Australia, summer rainfall zones are located in the northern half of the continent, including the northern part of Western Australia, the Northern Territory, Queensland and northern parts of New South Wales (Figure 3). With the exception of New South Wales, there is little to no sheep industry in these areas (Figure 4). In Australian summer rainfall zones, the most important gastrointestinal nematodes are *H. contortus*, *O. columbianum* and *T. colubriformis*, whilst other species of *Trichostrongylus* as well as *Teladorsagia* and *Nematodirus* species are regarded to be of secondary importance [16]. *Haemonchus contortus* is the most pathogenic species, mainly affecting sheep between 3–7 months of age, with peak infections typically occurring during the summer and autumn months [10].

Based on early research [28,30-32], adult populations of *H. contortus* and FECs increase during spring, peak in summer and early autumn, and then decline during winter [10]. Infective larval populations on pastures peak during January and February decline from March to August and reach their lowest levels around September to November [31]. From the beginning of autumn, an increasing proportion of larvae arising from eggs deposited during the summer and autumn months accumulate as arrested larvae in sheep of all ages, to resume their activity in the following spring [10].

Due to its pathogenicity, *O. columbianum* would rank second after *H. contortus*, but its prevalence has declined and in some areas, this species appears to have disappeared [10]. Concurrently, *O. venulosum*, an essentially non-pathogenic species, appears to have increased in prevalence. Precise reasons for the decline of *O. columbianum* are uncertain, but Donald *et al*. [10] suggested that possible factors might include the increasing use of effective anthelmintics, the cold-tolerance of its free-living stages compared with other species, such as *H. contortus*, and the increasing use of sown pastures, which is suspected to affect the establishment rate of *O. columbianum*. Dash [33] showed that in mixed infections, *O. venulosum* out-competes *O. columbianum*, which might be a factor contributing to the decline of the latter species. The infection pattern of *O. columbianum* in Australian summer rainfall zones is similar to that of *H. contortus*. However, the difference is that the larval stages of *O. columbianum* do not emerge from nodules in the large intestinal wall until winter [16].

Of the three most important small intestinal *Trichostrongylus* spp. in Australia, *T. colubriformis* predominates in summer rainfall zones of Australia [20]. Pasture contamination with larvae of *Trichostrongylus* spp. peaks during summer, and decreases in mid-winter and late spring [10]. In contrast to *H. contortus* or *Teladorsagia* species, burdens of *Trichostrongylus* in spring-born lambs increase throughout the summer, autumn and winter periods, to reach a maximum in the following spring at approximately 12 months of age. Burdens then decrease in the second summer due to the development of host resistance [10].

*Teladorsagia* spp. (represented by three morphotypes *Te. circumcincta*, *Te. trifurcata*, and *Te. davtiani*) [34] are commonly found, but their pathogenicity is difficult to estimate. In summer rainfall environments of Australia, infective L3 of *Teladorsagia* are most abundant on pastures grazed by spring-born lambs. Worm burdens follow closely the trend of larval availability, with peaks during summer, then declining during autumn to early winter [31].

Heavy infections with *Nematodirus* spp. (i.e., *N. spathiger* and *N. filicollis*) have been reported in lambs [35] but seldom present a serious problem. Other, less common species are *Strongyloides papillosus* and *C. ovina*, of which the latter occurs in areas bounding the southern limits of summer rainfall zones [10].

### Winter rainfall zones

Australian winter rainfall zones are in the southern part of the continent, including Tasmania, most parts of Victoria and the southern parts of Western Australia, Southern Australia and New South Wales (Figure 3). The highest sheep densities are found in western Victoria, south-western Western Australia and south-eastern New South Wales (Figure 4).

The most important causes of parasitic gastroenteritis in winter rainfall zones are *Te. circumcincta* and *T. axei* in the abomasum, and *T. colubriformis* and *T. vitrinus* in the small intestine, of which the latter species has the highest pathogenicity [21] and predominates in this environment [25,29]. In the Australian winter rainfall zones, these parasites are associated with death in weaners and productivity losses through all classes of sheep [10]. *Haemonchus contortus* infection appears to be confined to periods/regions experiencing higher than average summer rainfall [10] – or to spring and autumn. *Chabertia ovina* and *Nematodirus* spp. have been reported to be common throughout the winter rainfall zones, but these parasites are seldom considered to be a primary cause of disease or lost production [10]. The prevalence of infection depends on the availability of infective L3 on the pasture, which generally follows the trend of rainfall, with peaks in late winter, decreasing in spring to lowest levels in summer months. However, this pattern is modified by local spatial/temporal variations in weather [10].

In spring-born lambs, weaned between December and January, worm burdens are low during summer but increase during autumn when large numbers of L3 become available following the onset of rain [29,36]. In autumn-born lambs, weaned in August, high levels of infection build up in late winter and spring; these animals are prone to carry much larger worm burdens during their first summer than do spring-born lambs [37]. Worm burdens tend to decline in the following autumn, with the development of resistance as these sheep reach 12 months of age [10]. Worm burdens in adult sheep appear to be relatively constant throughout the year, with the exception of breeding ewes, in which a marked peri-parturient rise can occur [10].

In sheep of all age groups, infective L3s of *Te. circumcincta* ingested in winter, spring and early summer undergo, within the host, arrested development at the early fourth stage and resume their development in the following summer and early autumn. This situation can lead to enhanced pathogenic effects of the parasite at times of nutritional stress and might replenish adult worm populations at times when larval intake from pasture is low [37,38]. FEC patterns of sheep in this climatic zone consistently show a marked increase during summer and early autumn. This finding is reflected in the study of Anderson [36] who observed a negative correlation between the intake of infective L3s and FECs. Highest FECs were observed during summer, when levels of larval intake were low. Low FECs, on the other hand, were observed during autumn when there were increased levels of L3-contamination on pastures.

### Uniform rainfall zones

The non-seasonal or uniform rainfall zone in Australia is mainly found in the central and the south-eastern parts of New South Wales. The most important nematode genera occurring in this zone are *Teladorsagia* and *Trichostrongylus*[10]. In this environment, *T. colubriformis* and *T. vitrinus* both occur but at different times of the year. *Trichostrongylus colubriformis* mainly infects sheep during autumn, whereas the prevalence of *T. vitrinus* is higher in winter and spring [39]. In contrast, sheep in the arid regions of western New South Wales are predominantly infected with *T. rugatus*. These differences in temporal and spatial occurrence are likely determined by the varying environmental needs of their free-living stages [20]. *Haemonchus contortus* is common in uniform rainfall zones, but is usually present in moderate numbers and restricted to areas or periods with a wet summer climate [10]. Other common, but less important gastrointestinal nematodes occurring in this zone are *Nematodirus* spp. and *O. venulosum*[16]. The seasonal pattern of parasitic gastroenteritis in the uniform rainfall zone is largely a composite of patterns described for the summer and winter rainfall zones [10].

### Central role of diagnosis for studies of the epidemiology of parasites

The diagnosis of gastrointestinal nematode infections plays a major role in investigating parasite epidemiology. The *ante mortem* diagnosis of strongylid nematode infections in livestock has been based on the detection or enumeration of nematode eggs or larvae in the faeces by microscopic examination using the methods of flotation (e.g., McMaster chamber) and/or larval culture [40], although lectin staining of eggs has been evaluated for some species [41]. The larval culture method is time-consuming (taking 1–2 weeks for eggs to hatch and larvae to develop to L3s, depending on conditions) and requires an experienced microscopist to identify and distinguish L3s. In addition, the method of larval culture can be unreliable for establishing the relative abundance of eggs from different species present in a faecal sample, as their rates of survival and development can vary, depending on culture conditions [42].

*Post mortem* examination involves conducting a “total worm count”, followed by the morphological identification of adult and/or larval stages collected from the gut contents [40]. Importantly, the numbers of strongylid eggs per gram (EPG) of faeces do not necessarily correlate with the number of nematodes present in the gastrointestinal tract of a host, as adult females differ in their biotic potential/fecundity within and among species. This is particularly the case for nematodes with relatively low reproductive potential, such as species of *Trichostrongylus*[43]. In contrast, for species that are prolific ‘egg layers’, such as *Haemonchus contortus*, there is a relationship between EPG and the number of adult nematodes present in the host gut [44,45]. Importantly, some strongylids of ruminants, such as *H. contortus*, *T. axei*, and *Teladorsagia* and *Nematodirus* species, undergo arrested development (hypobiosis) at the larval stage in the host, when conditions in the external environment are unfavourable for parasite development and survival [46,47], in which case eggs are not produced by worms and excreted in the faeces.

Serological and immunological (e.g., coproantigen detection) approaches have also been evaluated for the specific diagnosis of infections. However, cross-reactivity with antigens of related parasites can occur, reducing the specificity of a diagnostic test [48,49]. A significant limitation of serological approaches is that they are not able to distinguish reliably between current and recent infections. The limitations of these diagnostic approaches have, to some extent, hampered progress in understanding the epidemiology of particular species of strongylid nematodes.

### The anthelmintic resistance problem and the need for molecular detection methods

Following the introduction of phenothiazines in the 1950’s, the control of gastrointestinal parasites has been achieved using chemical anthelmintics [50] and predominantly relies on the treatment with broad-spectrum parasiticides belonging to three main chemical classes: the benzimidazoles (BZ), the macrocyclic lactones (ML) and the imidazothiazoles/tetrahydropyrimidines (LV) [51] (Table 3). Although some anthelmintics, including derquantel (spiroindole) [52,53] and monepantel (an amino-acetonitrile derivative, AAD) [54], have been developed, success in the discovery of novel anthelmintics has been limited over the last two decades [4].

**Table 3:** Major classes of anthelmintics used for the treatment of nematode infections in livestock; their mode of action (if known) and proposed mechanisms of resistance

**Anthelmintic class**	**Understood mode of action**	**Proposed mechanisms of resistance**	**Reference**
Benzimidazoles	Bind to β-tubulin and prevent the formation of microtubules. Causes the inhibition of glucose uptake, protein secretion and microtubule production, leading to starvation of the parasite.	Mutations in the β-tubulin gene, causing structural changes in β-tubulin. As a consequence, the drug can no longer bind to its target site.	[93-95]
Imidazothiazoles/tetrahydropyrimidines	Mimic the action of acetylcholine causing spastic paralysis of the worms. Paralyzed worms are expelled by normal gut peristalsis, leading to rapid removal of present worms.	Poorly understood; possible involvement of structual changes in the nicotinic acetylcholine receptor, preventing the binding of the drug. Also proposed have been changes in the sensitivity of the receptor towards acetylcholine, which can lead to a cross-resistance with organophosphates.	[96-99]
Macrocyclic lactones (avermectins/milbemycins)	Causes an opening of glutamate-gated chloride channels (GluCI). This leads to an increased CI-ion influx into nerve cell, causing flaccid paralysis of the worm.	Poorly understood; possible involvement of: mutations in P-glycoprotein gene could cause a gain-of-function, leading to a more rapid removal of the drug from the parasite. Selection at glutamate- and γ-aminobutyric-acid gated chloride channels.	[100-102]
Amino-acetonitrile derivatives	The hypothesized mode of action involves a nematode-specific clade of acetylcholine receptor subunits.	Full or partial loss of the gene which encodes the particular type of acetylcholine receptor.	[54]

The frequent and often excessive use of these drugs has led to widespread problems with anthelmintic resistance in parasites of livestock [55]. Such resistance has emerged as a major bionomic and economic problem worldwide, being currently most severe in parasitic nematodes of small ruminants [56-58]. For instance, in Australia, it has been proposed that the prevalence and extent of resistance to all major classes of broad-spectrum anthelmintics is so widespread that it compromises parasite control and threatens the profitability of the sheep industry [51].

Given widespread anthelmintic problems, monitoring the drug-susceptibility and resistance status in different species of strongylid nematodes must be a high priority, and should be an important component of integrated management strategies. A number of methods, such as faecal egg count reduction test (FECRT) and egg hatch- and larval development assays, have been used for estimating levels of drug-susceptibility/resistance in strongylid nematodes of small ruminants and other livestock [59]. Most recent advances in the diagnosis of anthelmintic resistance have focused on the implementation of a standardized protocol for the egg hatch test [60], and the development and standardization of a larval migration inhibition test [61]. However, such assays can be laborious and time consuming to perform, may suffer from a lack of reliability, sensitivity and reproducibility of results [55]. Clearly, there has been a need to develop improved diagnostic methods using molecular technology. Although there have been some advances, the definition of genetic markers for use in molecular assays for the accurate detection of resistance has been a major challenge, mainly because the mechanisms of resistance in parasitic nematodes are still unclear for many drugs [62].

Molecular methods have been proposed to provide new alternatives to commonly applied *in vivo* and *in vitro* techniques for the diagnosis of anthelmintic resistance, and may be able to overcome some of their limitations [56,61-63]. Crucial for the development of molecular diagnostic assays is knowledge of the mechanisms linked to resistance and reduced susceptibility in parasites [5,56,62].

BZ resistance in nematodes seems to be best understood at the molecular level, whilst much less is known about resistances against other classes of anthelmintics [55]. Originally, a single nucleotide polymorphism (SNP) at codon 200 of the beta-tubulin isotype 1 was believed to be linked to BZ resistance [5,64] and has been demonstrated in resistant strains of *H. contortus*[65], *T. colubriformis*[66] and *Te. circumcincta*[67] in sheep. At least two more SNPs at codons 167 and 198 have been detected, but appear to be less common in different species of trichostrongylid nematodes [5,62]. Recent work has also suggested a link to the drug transporter P-glycoprotein, proposed to play a role in the transport of an anthelmintic away from its site of action and may also select for resistance to MLs [62].

Allele-specific PCRs were developed to determine the BZ-resistance genotype of adult worms of *H. contortus*[64] and *Te. circumcincta*[67]. This work was extended by combining the previously described PCR assays with an RFLP method [68], which allowed the phenetic characterisation and identification of L3s of *H. contortus*, *T. colubriformis* and *Te. circumcincta*. Alvarez-Sanchez *et al*. [69] designed a real-time PCR (RT-PCR) assay to assess the frequency of the beta-tubulin isotype 1 allele (linked to codon 200) in nematode samples. As stated by the authors, the diagnosis of BZ resistance using this test showed an agreement with phenotypic tests, including the egg hatch test and the faecal egg count reduction test [56]. In contrast to BZ resistance, the molecular mechanisms linked to other resistances (e.g., to LVs and MLs) are not yet well understood. Indeed, the mechanisms of these resistances are likely to be complex and multigenic [62,70].

In spite of progress on the molecular biology of resistance, there is no practical molecular test for the detection of multiple resistances in multiple species of gastrointestinal parasites of small ruminants. In addition, all currently employed molecular assays used infective L3s (requiring the culturing of eggs for 1–2 weeks) or adult nematodes (only available through necropsy of the host), but none of them has been evaluated for the detection of anthelmintic resistance directly in mixed populations of nematode eggs. A practical molecular method for multi-drug resistance detection in nematode eggs would be a major advance.

### Improved molecular tools for the specific diagnosis of nematode infections and detection of anthelmintic resistance in particular parasites in combination with conventional methods

Although the diagnosis of drug resistance has major challenges, there have been significant advances in the specific molecular diagnosis of nematode infections. PCR-based methods using specific genetic markers in the internal transcribed spacers of nuclear rDNA [71] have provided enhanced epidemiological tools. For instance, some of our recent studies [72-75] have demonstrated that real-time PCR (RT-PCR) and multiplexed-tandem PCR (MT-PCR) assays can replace the inaccurate and time-consuming method of larval culture. The high throughput method of MT-PCR [73] takes less than 1 day to perform, compared with at least a week for conventional larval culture, thus reducing the time that the farmer has to wait for a diagnosis. This molecular platform does not require detailed parasitological training or molecular knowledge on the part of the operator, has high sensitivity and specificity, and has broad applicability, in that it can be used to conduct large-scale epidemiological studies and to support the diagnosis of drug resistance in combination with conventional FECRT [75]. Importantly, the MT-PCR platform delivers, rapidly, objective and detailed results to a generic or specific level, which is of major value for enhanced control.

The MT-PCR established by Roeber *et al*. [73] essentially meets the international standards [76,77] for routine use in a laboratory setting for research or routine diagnostic purposes and has some significant advantages over traditional methods, particularly regarding the interpretation of FEC results and recommendations about anthelmintic treatment. This test improves the diagnosis of infections with nematode species, which are problematic to detect or identify by traditional coprological techniques, either because of their morphological/morphometric similarity with other species/genera (i.e., *Teladorsagia* and *Trichostrongylus*, *C. ovina* and *O. venulosum*) or their unfavourable development under ‘standard’ culture conditions (27°C). Current evidence indicates that this MT-PCR assay is highly adaptable and applicable to large-scale epidemiological studies as well as the detection and monitoring of drug resistance in gastrointestinal nematodes when combined with the use of FECRT.

## Conclusion

Given the economic impact of gastrointestinal nematodes of sheep and other small ruminants in the livestock industry in Australasia and other countries, over the years, there has been a major focus on investigating the biology and epidemiology of these parasites and on improving treatment and control. However, the spread of anthelmintic resistance [4,5] has led to an increased need for strategic and integrated control of parasites, with enhanced management components and reduced anthelmintic use [50]. With this focus comes an increased need to explore and understand the epidemiology (e.g., prevalence, distribution, and seasonal patterns of transmission and disease in different climatic zones) of different species of gastrointestinal nematodes and of anthelmintic resistance employing practical and reliable molecular diagnostic techniques. Recent studies [73-75] have delivered advanced PCR tools to underpin epidemiological studies and to support investigations into the nature and extent of resistance, in combination with conventional parasitological techniques (e.g., FECRT). These PCR tools were developed to overcome the constraints of traditional methods that have not undergone enhancements for many years. In contrast to traditional techniques, these tools now allow detailed studies of individual nematode species and genera, not previously possible, and provide a solid basis for epidemiology studies. In future, these methods, and high-performance field-based assays and high-throughput sequencing technologies provide prospects for better disease surveillance within the context of integrated parasite control programs and for shorter response times to tackle and control nematodiases (as diagnosis using molecular techniques usually takes less time compared with culture- or microscopy-based approaches) and, ultimately, to provide greater protection for the consumer of animal products through reduced anthelmintic use. Epidemiological data obtained using such tools would help authorities, such as the Office International des Epizooties (OIE), Food and Agricultural Organization (FAO), in the detailed mapping of the prevalence and distribution of parasites and drug resistance, make forecasts about the occurrence and spread of disease, in order to implement appropriate contingency plans and guidelines for the effective and sustainable control of nematodiases. In the present revolution in genomics, bioinformatics and the development of molecular tools (e.g., [78-103]), many technological advances are expected, some of which should enhance our ability to understand and tackle parasitic diseases of animals.

## Competing interests

The authors declare that there are no competing interests.

## Authors’ contributions

FR and RBG drafted the manuscript, with inputs from ARJ. FR prepared the Tables and Figures. All authors read and approved the final version of the manuscript.
